# Current state and future potential of radiopharmaceutical therapy in breast cancer

**DOI:** 10.1016/j.breast.2026.104946

**Published:** 2026-10-17

**Authors:** Joyce O'Shaughnessy, Neil K. Taunk, Gary A. Ulaner

**Affiliations:** a Texas Oncology, Baylor University Medical Center, Sarah Cannon Research Institute, 3140 Worth Street, Suite 400, Dallas, TX, 75246, USA; b Abramson Cancer Center, Department of Radiation Oncology and Department of Radiology, Perelman School of Medicine, University of Pennsylvania, 3400 Civic Center Blvd, Philadelphia, PA, 19104, USA; c Hoag Family Cancer Institute, 16105 Sand Canyon Ave Ste 215, Irvine, CA, 92618, USA; d Keck School of Medicine, University of Southern California, Los Angeles, CA, USA

**Keywords:** Breast cancer, Radiopharmaceutical therapy, HER2, FAP, Multidisciplinary team

## Abstract

Radiopharmaceutical therapy has been adopted with success in malignancies such as prostate cancer and neuroendocrine tumors. This approach often combines a diagnostic agent to measure and assess target availability prior to administering a parenteral radiotherapeutic agent against the same target. Emerging evidence and ongoing research suggest that this approach could have a beneficial impact in breast cancer treatment, with a number of different breast cancer-specific or tumor microenvironment targets. There are several agents under investigation, with the majority in early-phase trials. This narrative review will provide a brief introduction to radiopharmaceutical imaging and therapy for breast cancer, discuss emerging therapeutic targets under investigation in breast cancer, and discuss challenges with the implementation of radiopharmaceuticals in breast cancer.

## Highlights

• There is a need for novel effective therapies in advanced breast cancer.
• Radiopharmaceuticals are emerging as a promising treatment for breast cancer.
• Many targets are being investigated for radiopharmaceutical breast cancer treatment.
• Multidisciplinary teamwork will help adopt radiopharmaceuticals in breast cancer.

## Graphical abstract

**Figure:**
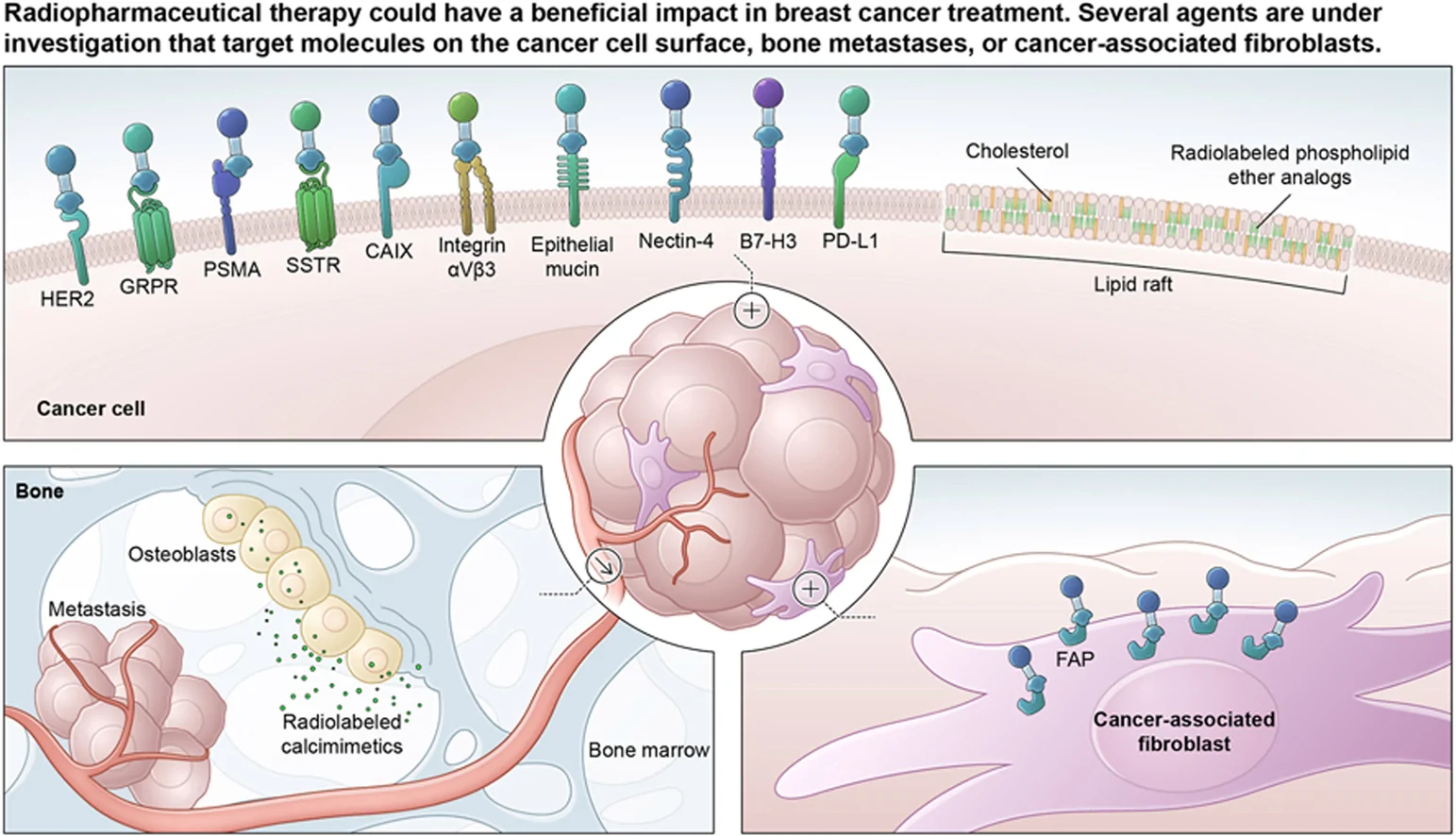

## 1 Introduction

In breast cancer (BC), the development of recurrent disease progression and treatment resistance, along with inter- and intratumoral heterogeneity, underscores the need for novel treatment classes that can improve survival and quality of life for patients [1]. Theranostics—a portmanteau of “therapy” and “diagnostics”—is a novel class of systemic therapy that can potentially address these treatment needs [2].

Theranostics comprise a pair of systemic radiopharmaceuticals using different radionuclides that target a single biomarker for both imaging (referred to as radiopharmaceutical imaging) and treatment (referred to as radiopharmaceutical therapy or “RPT”) applications (Fig. 1); in some cases, these radiopharmaceuticals target specific ligands within the tumor and are referred to as radioligand imaging and radioligand therapy, respectively [3]. The main advantage of the paired approach of theranostics is that it uses imaging-informed confirmation of target expression across multiple lesions before initiating treatment. Compared with conventional systemic treatments, theranostics allows precise targeting and delivery of cytotoxic radiation treatment based on known and potentially quantifiable target expression, potentially mitigating the imprecision of cytotoxic chemotherapy [4], [5]. The radiopharmaceuticals used for theranostics are made up of three essential components: a radionuclide with or without a chelator to hold the radionuclide, a linker if not directly labeled, and a targeting vector (ligand) [6]. This framework of linking a targeting ligand with a cytotoxic radioactive agent is similar in concept to that of antibody–drug conjugate (ADC) therapy. Ligands can be peptides, antibodies, or small molecules [7].

**Fig. 1:**
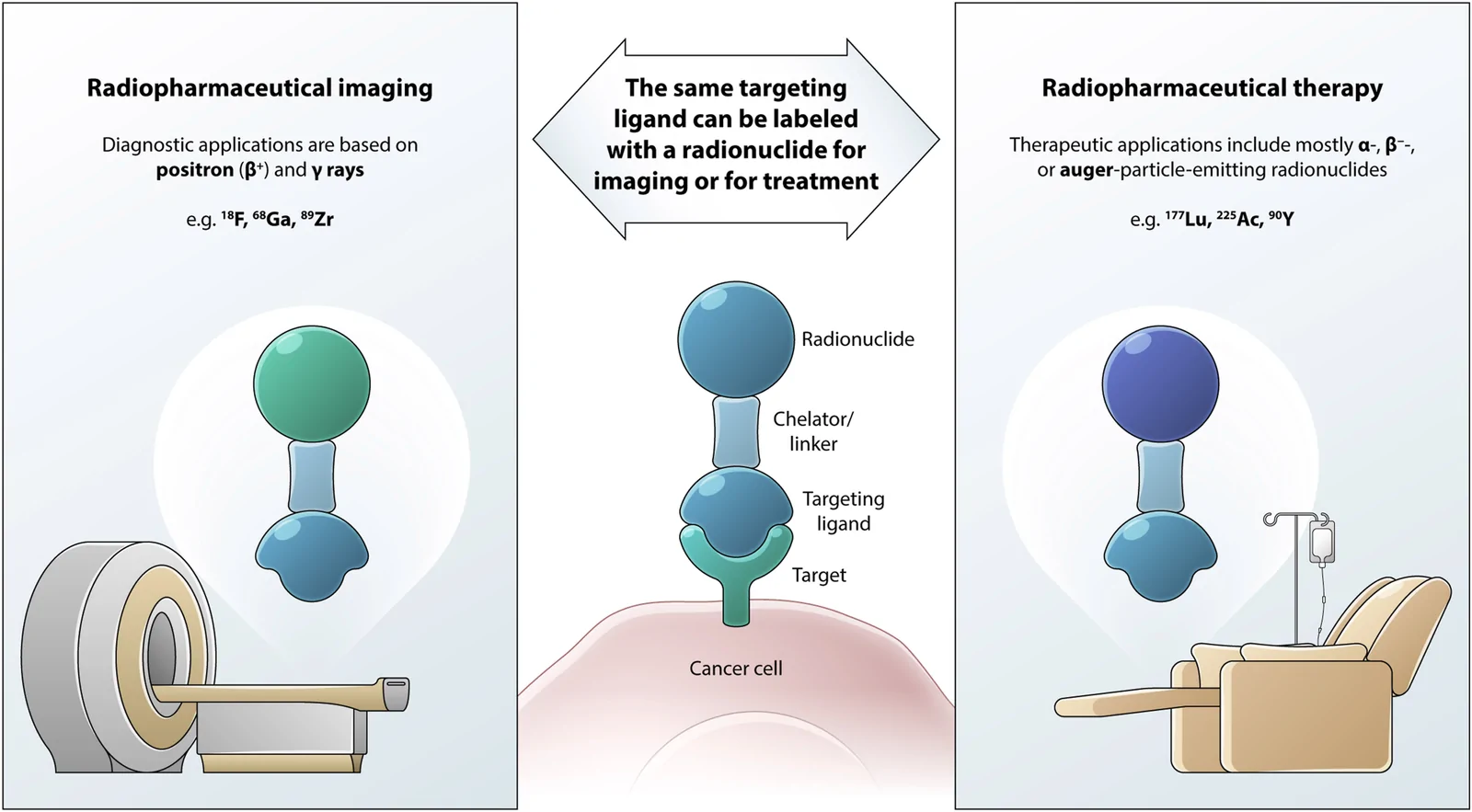
Theranostic goals of radiopharmaceuticals in breast cancer.

Radionuclides used for diagnostic imaging generally have short half-lives and emit either positrons (β^+^-particles) used for positron emission tomography (PET), e.g. fluorine-18 [^18^F] used in [^18^F]fluorodeoxyglucose-PET ([^18^F]FDG-PET) imaging, or photons (γ-radiation) used for single-photon emission computed tomography (SPECT), e.g. technetium-99m [^99m^Tc]), which interact minimally with surrounding tissue without discernible therapeutic effect [6], [8], [9], [10], [11]. The low linear energy transfer (LET) and deep tissue penetration (33–164 mm) of γ-radiation limit the therapeutic potential for delivering targeted cytotoxic radiation [5], [6], [12].

Radionuclides that emit β^–^-particles, α-particles, and/or Auger electrons are used for therapy, as they have a suitable range of half-lives, and have sufficient LET to effectively kill tumor cells [6], [11], [13]. β^–^-particles, the most frequent type of radionuclide used for radiopharmaceutical therapy, have low LET (0.1–2 keV/μm), long path lengths (1–12 mm), and attenuate their energy slowly, allowing energy deposition over a large tissue volume [5]. These properties make them potentially cytotoxic against large tumors, addressing intratumoral heterogeneity via their crossfire effect [6], [13], [14], [15], [16]. The crossfire effect, functionally analogous in outcome to the bystander effect observed with some ADCs, but mechanistically distinct, describes the phenomenon wherein radionuclides with a long radiation path length can irradiate neighboring tumor cells that do not express the target [4], [5]. α-particles have high LET (20–230 keV/μm), over a moderate path length of a few cell lengths (50–100 μm), suggesting that they could be effective for targeting micrometastases and hypoxic tumors, and generally limiting their potential crossfire capacity [6], [15], [16], [17], [18], [19]. Auger radiation releases low-energy electrons with high LET (∼4–29 keV/μM) over a very short distance (2–500 nm), killing single cancer cells [11], [15], [16], [20]. Additionally, certain radionuclides can emit more than one type of radiation energy, e.g. radionuclides that emit β- or γ-radiation can also emit Auger and conversion electrons (when γ energy is absorbed by an electron rather than being expelled) [21], [22]. Conversely, many β^–^-emitters and some α-emitters can also emit γ-rays, allowing for γ-camera, e.g. SPECT imaging (Table 1) [8], [23].

**Table 1:** Radionuclides being investigated for radiopharmaceutical diagnostic imaging in BC.

Radionuclide	Radiation type	Status of radiopharmaceuticals in BC	Half-life	Imaging modality	Also used for therapy	References
^125^I	Auger	Under investigation, i.e. ^125^I-FAPI-01; ^125^I-KX1	59.4 days	SPECT	Yes	[24], [25], [26]
^11^C	β^+^ (positron)	Under investigation, i.e. ^11^C-choline; ^11^C-tyrosine	20 min	PET		[13], [25], [27]
^64^Cu	β^+^ (positron); β^−^; Auger	Under investigation, i.e. ^64^Cu-DOTA-trastuzumab	12.7 h	PET		[24], [25], [28]
^18^F	β^+^ (positron)	In use, i.e.,^18^F-FDG; ^18^F-FES; ^18^F-NaF Under investigation, i.e. ^18^F-FAPI-74	110 min	PET		[10], [25], [29]
^68^Ga	β^+^ (positron)	Under investigation, i.e. ^68^Ga-FAPI-02; ^68^Ga-ABY-025	68 min	PET		[24], [25]
^124^I	β^+^ (positron)	Under investigation, i.e. ^124^I-trastuzumab; ^124^I-I2-PARPi	4.2 days	PET		[21], [25], [30]
^89^Zr	β^+^ (positron)	Under investigation, i.e. ^89^Zr-trastuzumab;^89^Zr-pertuzumab	78.4 h	PET		[21], [24], [25]
^131^I	Gamma; β^−^; Auger	Under investigation, i.e. ^131^I-trastuzumab; ^131^I-GMBI-anti-HER2-VHH1	8.0 days	SPECT	Yes	[21], [24], [25]
^111^In	Gamma	Under investigation, i.e. ^111^In-trastuzumab	67.2 h	SPECT		[21], [25]
^99m^Tc	Gamma	In use, i.e. ^99m^Tc-MIBI; ^99m^Tc-sestamibi Under investigation, i.e ^99m^Tc-FL-L3	6.0 h	SPECT		[13], [21], [25]

BC, breast cancer; PET, positron emission tomography; SPECT, single-photon emission computed tomography.

Radiopharmaceuticals have been proven to be effective in treating multiple solid tumor types, including metastatic prostate cancer and neuroendocrine tumors (NETs), demonstrating improved progression-free survival and overall survival (OS) in some indications [31]. Although theranostic pairs for imaging and therapy have not yet been approved for BC, this approach is currently under investigation in many clinical trials. This review will provide an overview of the current status of radiopharmaceutical agents in BC with increasing focus on therapeutic agents, highlighting the potential of this modality to offer personalized treatment potentially less subject to cross-resistance for patients with BC.

## 2 Radiopharmaceutical imaging in breast cancer

Radiopharmaceuticals are used for diagnostic imaging in BC as part of PET or SPECT functional imaging, and bone scintigraphy [6], [12], [13], [32]. This functional imaging may be combined with computed tomography (CT) and magnetic resonance imaging (MRI) to assess both the structure and biological activity of cancers, and is used for diagnosis, staging, treatment selection, and monitoring treatment response [9], [21], [33]. Radiopharmaceutical imaging has significant advantages over conventional cross-sectional imaging modalities (i.e. CT scan or MRI), including enhanced disease detection and staging via functional imaging, improved patient selection for targeted treatment, and predicting and monitoring treatment response [14], [30], [34], [35].

The first radiopharmaceuticals used to image primary and metastatic BC (MBC) included ^99m^Tc sestamibi (also known as ^99m^Tc-methoxy isobutyl isonitrile), sodium [^18^F]fluoride, and ^18^F-FDG [10]. Current guidelines from the National Comprehensive Cancer Network (NCCN), European Association of Nuclear Medicine (EANM), and Society of Nuclear Medicine and Molecular Imaging (SNMMI) recommend the use of ^18^F-FDG, a radioligand that detects glucose metabolism, for PET imaging to complete initial staging of BC (stages IIB, III, and IV), treatment planning, and monitoring treatment response [36], [37]. The guidelines also endorse the utility of PET in assessing early metabolic response in non-metastatic triple-negative BC (TNBC) and human epidermal growth factor receptor 2-positive (HER2+) BC. The phase 2 PHERGain trial demonstrated the utility of ^18^F-FDG in informing treatment planning by identifying patients with early-stage HER2+ BC who had complete metabolic response to pre-operative HER2-directed therapy alone, without cytotoxic therapy [38]. In MBC, ^18^F-FDG is widely used to monitor treatment response, and is particularly useful for assessing bone metastases, as bone-regenerating osteoblastic activity that confounds interpretation of bone scintigraphy is not ^18^F-FDG-avid. ^18^F-FDG imaging is also useful in detecting metastatic disease at presentation as well as recurrent disease, especially in the context of equivocal findings on conventional imaging [39]. PET imaging with ^18^F-FDG is generally not recommended for patients with invasive lobular carcinoma due to lower ^18^F-FDG avidity, while the alternative tracer [^18^F]fluoroestradiol (^18^F-FES) may provide greater accuracy in staging invasive lobular carcinoma [39].

In 2020, ^18^F-FES became the first BC-specific radiopharmaceutical diagnostic approved for use with PET imaging for the detection of estrogen receptor (ER)-positive metastases [40]. The use of ^18^F-FES is recommended in recent guidelines for BC staging, including diagnosis of suspected metastatic disease that is difficult to biopsy, and for assessment of ER expression to enable patient selection for endocrine therapy (ET) [41], [42].

## 3 Potential for radiopharmaceutical therapy in breast cancer

There are multiple different classes of effective anticancer agents in BC. These include hormone therapies (e.g. aromatase inhibitors), targeted treatments (e.g. cyclin-dependent kinase type 4 or 6 inhibitors), cytotoxic chemotherapy (e.g. paclitaxel), and ADCs, among others [36]. Radiopharmaceutical therapy may represent a future approved class of agents. The approval and adoption of radiopharmaceutical therapy in patients with somatostatin receptor-positive (SSTR+) NETs, and in men with prostate-specific membrane antigen-positive (PSMA+) metastatic androgen pathway modulation-resistant prostate cancer, have engendered interest in applying this modality to MBC [43], [44]. The therapeutic effect of radiopharmaceuticals is derived from the targeted delivery of cytotoxic radiation to tumors, largely via direct tumor cell DNA damage [4], [5]. Radionuclides with a longer tissue penetration range, generally β^–^-emitters, can damage surrounding cancer cells through a crossfire effect, eliminating the need for the radiopharmaceutical to bind to every cell in the tumor mass [4]. Therapy agents must still be pharmacokinetically optimized for the different ligands to allow for rapid washout or lack of uptake in non-target tissue, and high retention and uptake in target tissue. This is especially useful in BC, which often has high inter- and intratumoral target expression heterogeneity [11]. Compared with other systemic treatments, radiopharmaceutical therapy is also less likely to be thwarted by compensatory cell-signaling networks that are activated in BC to bypass the cytotoxic effects of chemotherapy and targeted molecular therapies [5]. While the cytotoxicity of the radioactive payload depends on ligand binding of the radiopharmaceutical agent and tumor target expression, the therapeutic effect is not due to interruption of cellular signaling [45]. Importantly, the target itself may not be an oncogenic driver and successful engagement of the target may not lead to anti-proliferative or pro-apoptotic effects. Rather, ligand binding of the radiopharmaceutical delivers the radionuclide to the tumor cell, damaging DNA and essential proteins needed for survival.

## 4 Targets and radiopharmaceutical therapies for breast cancer

The goal of radiopharmaceutical therapy is to identify and treat patients whose cancers express tumor-specific molecular targets, while limiting off-target toxicity to normal tissue [6], [35]. Below, we will discuss radiopharmaceutical therapy targets under investigation in MBC (Fig. 2 and Table 2), highlighting the potential of this novel therapeutic modality. While several basket trials have reported anti-tumor activity or safety in studies that included patients with BC, the majority of BC-specific trials are active or pending reporting results. Available data are generally limited to early-phase trials and small sample cohorts or case studies, and these preliminary results should be interpreted with caution.

**Fig. 2:**
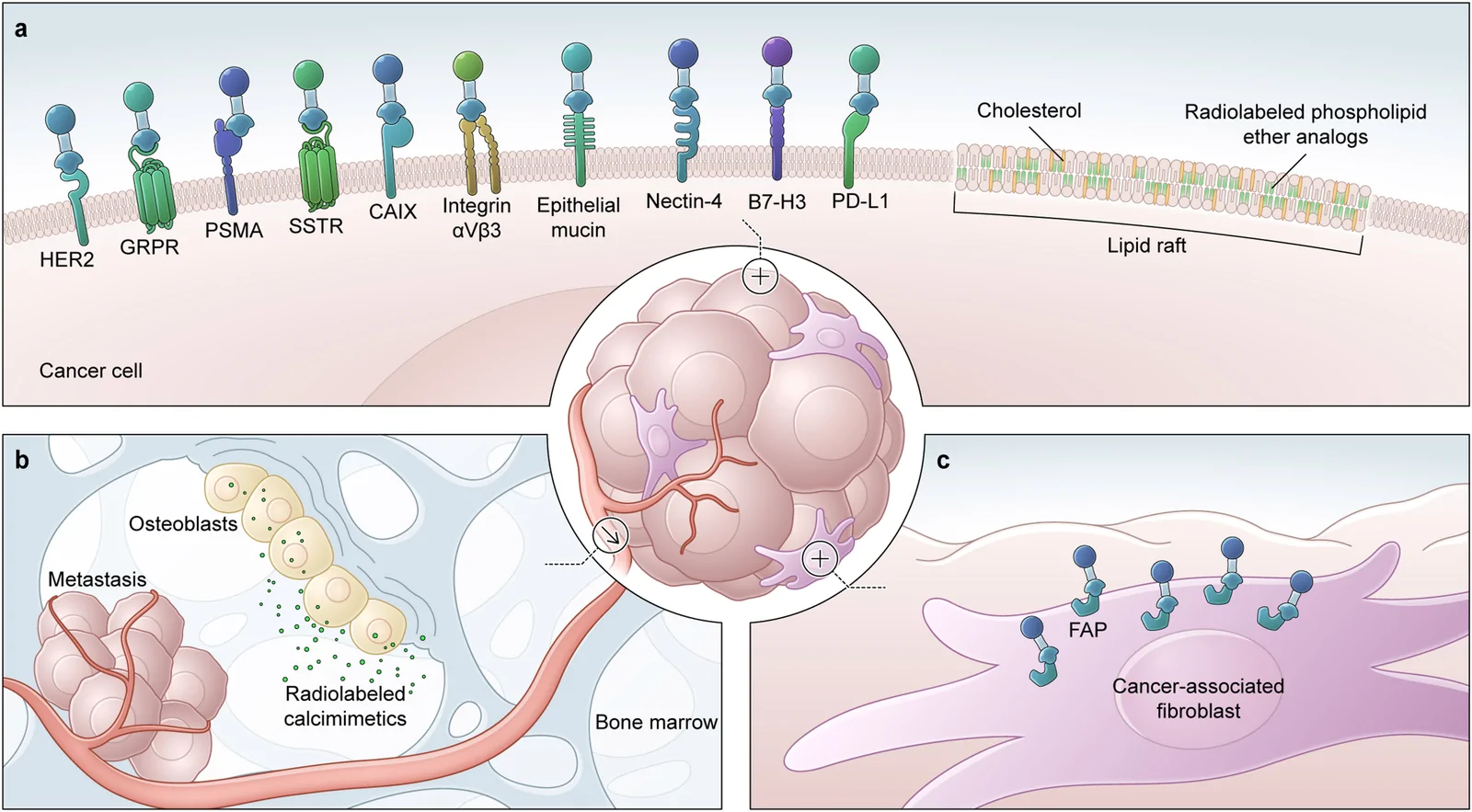
Targets under investigation for breast cancer radiopharmaceutical therapy. a) Radiopharmaceutical therapy targets on the cell surface of cancer cells. b) Calcimimetic radiopharmaceutical therapies mimic the action of calcium and are incorporated into the bone matrix. c) FAP is a target of radiopharmaceutical therapy that is expressed on cancer-associated fibroblasts. CAIX, carbonic anhydrase IX; FAP, fibroblast activation protein; GRPR, gastrin-releasing peptide receptor; HER2, human epidermal growth factor receptor 2; PD-L1, programmed death-ligand 1; PSMA, prostate-specific membrane antigen; SSTR, somatostatin receptor.

**Table 2:**
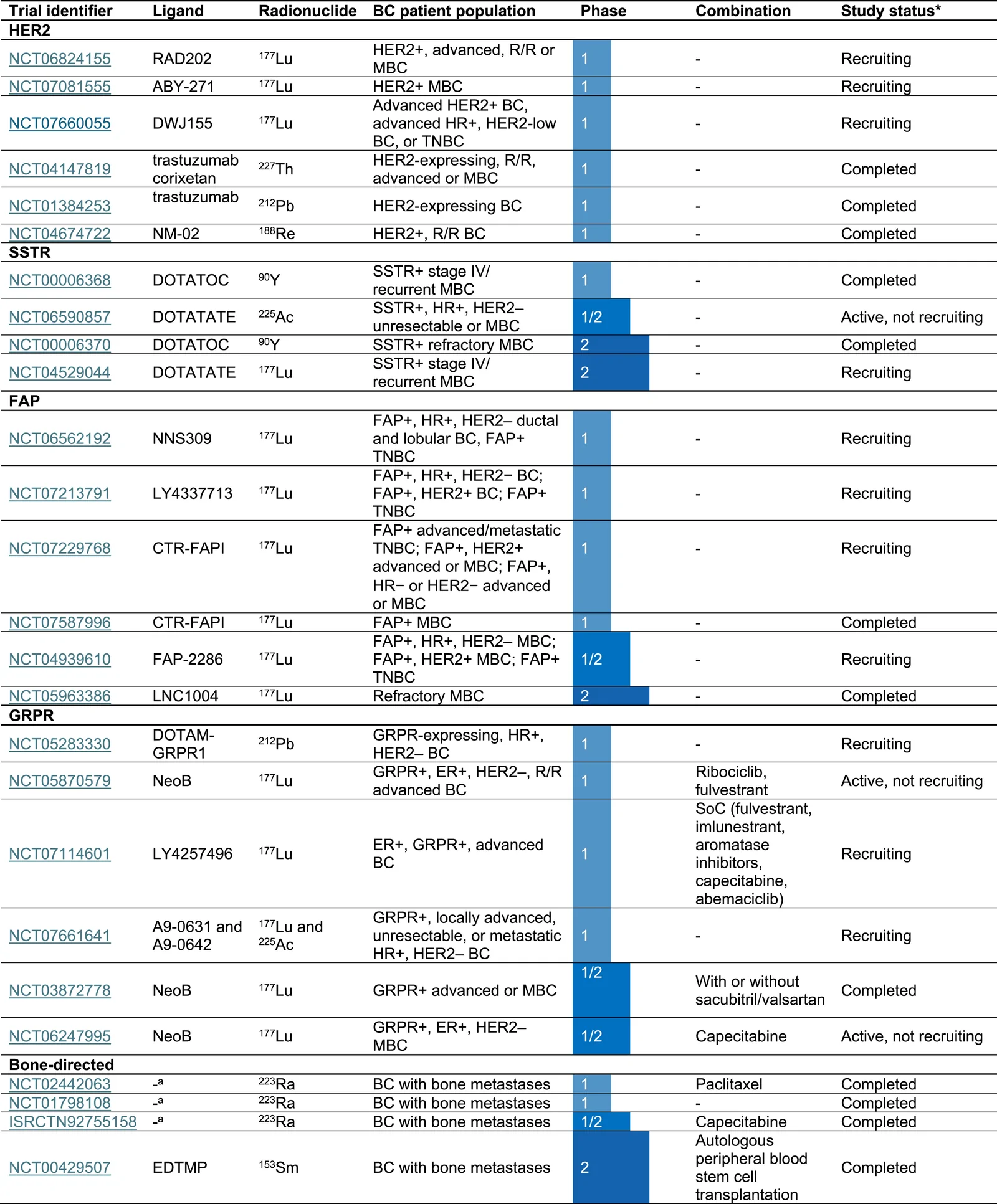

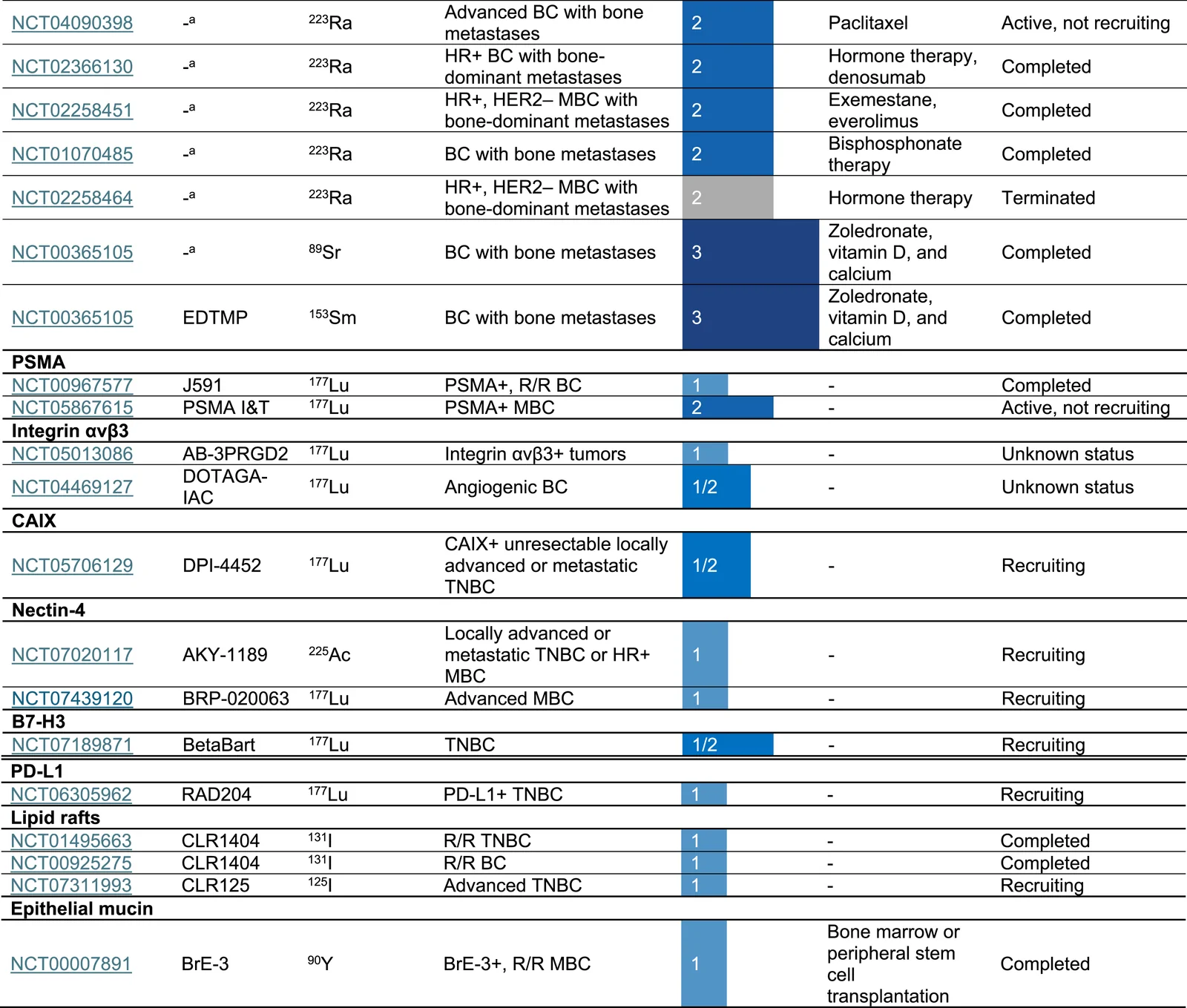
Clinical trials investigating radiopharmaceutical therapies in BC.

^a^These compounds do not have a ligand, as the radionuclide itself served as calcimimetic. *Status as of August 28, 2026. BC, breast cancer; CAIX, carbonic anhydrase IX; CAIX+, CAIX-positive; EDTMP, ethylenediamine tetra (methylene phosphonic acid); ER, estrogen receptor; ER+, ER-positive; FAP, fibroblast activation protein; FAP+, FAP-positive; GRPR, gastrin-releasing peptide receptor; GRPR+, GRPR-positive; HER2, human epidermal growth factor receptor 2; HER2+, HER2-positive; HER2–, HER2-negative; HR+, hormone receptor-positive; HR–, hormone receptor-negative; I&amp;T, imaging and therapy; integrin αvβ3+, integrin αvβ3-positive; MBC, metastatic BC; PD-L1, programmed death-ligand 1; PD-L1+, PD-L1-positive; PSMA, prostate-specific membrane antigen; PSMA+, PSMA-positive; R/R, relapsed or refractory; SoC, standard of care; SSTR, somatostatin receptor; SSTR+, SSTR-positive; TNBC, triple-negative BC.

### 4.1 Human epidermal growth factor receptor 2 (HER2)

HER2 is a receptor tyrosine kinase that plays a key role in regulating cell growth, survival, and differentiation of BC due to signaling dysregulation via gene amplification or acquisition of a somatic activating mutation [46], [47]. HER2 has long been recognized as a critical therapeutic target in BC, where it is associated with prognosis and treatment response [46], [48]. Approximately 15–20% of BCs have high HER2 gene amplification and protein expression; additionally, approximately 60% of those traditionally classified as HER2-negative have low levels of HER2 protein expression and can benefit from HER2-targeted ADCs [49], [50], [51], [52]. Treatment with anti-HER2 therapies, while generally highly effective, is associated with treatment-limiting toxicities such as cardiac toxicity, interstitial lung disease, myelosuppression, and neuropathy [53].

Radiopharmaceutical therapies that target HER2 have the potential to address resistance to standard HER2-directed therapies [54]. Two clinical trials are investigating the β^−^-emitting radionuclide lutetium-177 (^177^Lu) with the single-domain antibody RAD202 (NCT06824155) and the affibody (a small engineered protein that mimics an antibody) ABY-271 (NCT07081555) in patients with HER2+ MBC. While results from the first-in-human study with ^177^Lu-RAD202 are not yet available, preclinical results in HER2+ xenografts showed reductions in tumor volume, while ^177^Lu-ABY-271 showed favorable biodistribution and potent antitumor activity in HER2+ murine xenograft models [55], [56].

Three other trials are treating HER2+ MBC with a trastuzumab-derived antibody conjugated to thorium-227 (^227^Th; BAY2701439; NCT04147819), lead-212 (^212^Pb; NCT01384253), or rhenium-188 (^188^Re; NCT04674722), and findings are awaited. Finally, patients with advanced HER2+ BC, advanced hormone receptor-positive (HR+), HER2-low BC, or TNBC are being recruited to an open-label, phase 1 trial (NCT07660055) that will be investigating the safety, tolerability, dosimetry, and preliminary activity of the β-emitting, HER2-targeted radiopharmaceutical [^177^Lu]Lu-DWJ155.

### 4.2 Somatostatin receptor (SSTR)

The peptide somatostatin inhibits neuroendocrine actions across various organs affecting the exocrine, endocrine, and central nervous systems, preventing hormone secretion, angiogenesis, and cell proliferation [57], [58]. Somatostatin also has a role in carcinogenesis as its dysregulation in pathophysiologic conditions can prevent tumor suppression and anti-proliferative effects [58], [59]. The SSTR may be expressed in tumor cells as well as in the tumor microenvironment (i.e. tumor-associated macrophages), most commonly in NETs, including in up to 70% of patients with neuroendocrine BC, and roughly 30% of patients with ER-positive MBC [60], [61], [62], [63], [64]. Somatostatin analogs are the standard of care to treat NETs that express SSTR and have been shown to inhibit tumor cell growth via SSTR type 2 [65], [66]. In the pivotal phase 3 NETTER-1 trial, the radiopharmaceutical peptide ^177^Lu-DOTA-(Tyr3)-octreotate (^177^Lu-DOTATATE), which binds to the SSTR, plus high-dose octreotide, a somatostatin analog, showed clinically meaningful improvements in survival and quality of life vs high-dose octreotide alone in patients with advanced SSTR + midgut NETs [67], [68].

SSTR type 2 expression is present in all BC subtypes (up to 98%), but is more prevalent in HR+ disease (up to 100%) [69], [70], [71]. Most patients with HR + MBC develop ET-resistant disease, and SSTR-directed therapies are being developed in these patients to provide an alternative to chemotherapy [72], [73], [74], [75]. Multiple radiopharmaceuticals that utilize somatostatin analogs as ligands are currently being evaluated for the treatment of SSTR + MBC, including ^177^Lu-DOTATATE (NCT04529044), yttrium-90-DOTA-(Tyr3)-octreotide (^90^Y-DOTATOC; NCT00006370, NCT00006368), and actinium-255-DOTA-(Tyr3)-octreotate (^225^Ac-DOTATATE; NCT06590857). Case reports describe a patient with SSTR + MBC with neuroendocrine differentiation who had a biochemical complete response following treatment with ^90^Y-DOTATOC, a patient with primary neuroendocrine breast carcinoma who had a partial response with ^177^Lu-DOTATOC, and another heavily pretreated HR+, SSTR + patient who had a near complete response after two cycles of ^225^Ac-DOTATATE [64], [76], [77].

### 4.3 Fibroblast activation protein (FAP)

Fibroblast activation protein (FAP) is a type II integral membrane glycoprotein with low expression in normal tissue and high expression in cancer-associated fibroblasts (CAFs), the major subpopulation of cells in the stroma of most epithelial cancers [78], [79], [80], [81], [82], [83]. FAP-targeted radiopharmaceuticals bind to FAP-expressing CAFs, killing the CAFs as well as neighboring tumor cells via crossfire radiation [84]. CAF depletion can also enhance the efficacy of concomitant cytotoxic and targeted anticancer agents by reducing CAF-associated fibrosis, allowing for increased drug penetration [85]. Similarly, reducing CAF-associated immunosuppression through CAF depletion can enhance the activity of immunotherapeutic agents [86]. Ongoing clinical trials of FAP-targeted radiopharmaceutical therapies utilize FAP-targeted ligands (i.e. peptides) or small-molecule inhibitors of FAP.

Findings in 12 patients with FAP-expressing advanced adenocarcinomas, including MBC, have shown that the FAP peptide radiopharmaceuticals ^177^Lu-rofapitide tetraxetan (^177^Lu-FAP-2286) and ^177^Lu-DOTA-S-amide-FAP inhibitor (^177^Lu-DOTA.SA.FAPi) led to high tumor uptake and retention, with no significant adverse events (AEs) [87], [88]. Similar results were reported with gallium-68-rofapitide tetraxetan (^68^Ga-FAP-2286), and three of the four patients with MBC receiving ^177^Lu-FAP-2286 showed stable disease after 4 months of follow-up [89]. Likewise, treatment with the small-molecule FAP inhibitor ^177^Lu-FAPi-46 showed that three of five patients with FAP-expressing MBC had stable disease 6–8 weeks after treatment, with no notable toxicities. One study with the theranostic pair ^68^Ga-DOTA.SA.FAPi and ^177^Lu-DOTAGA.FAPi showed disease control in 18 of 19 patients with pretreated MBC, with acceptable toxicity, no grade 3–4 AEs, and no severe hematologic, kidney, or liver toxicities [90].

A phase 2 trial of the radiopharmaceutical small-molecule FAP inhibitor ^177^Lu-DOTA-Evans blue-FAP (^177^Lu-LNC1004) (NCT05963386) in pretreated patients with FAP-expressing metastatic carcinoma showed acceptable safety (the most common AE was hematotoxicity), with nearly half of patients achieving disease control associated with prolonged progression-free survival and OS in the overall cohort (n = 28), with one of three patients with MBC having stable disease [91]. Four phase 1 trials are investigating the efficacy, safety, and dosimetry of the β^−^-emitting FAP-targeted peptide ^177^Lu-NNS309 (NCT06562192), peptide ^177^Lu-Chinese tumor-targeting FAP inhibitor (^177^Lu-CTR-FAPi) (NCT07229768 and NCT07587996), and the cyclic peptide ^177^Lu-FAP-2286 (NCT04939610) in patients with FAP-expressing MBC [92]. These studies are using a theranostic imaging approach to confirm FAP expression with FAP-targeted PET. A case report showed that a patient with MBC who received chemotherapy in combination with ^177^Lu-FAP-2286 had a complete metabolic response with no grade 3–4 AEs during follow-up [93]. Another ongoing trial is evaluating the safety, tolerability, and dosimetry of the β^−^-emitting FAP-targeted radioligand LY4337713 (NCT07213791) in patients with FAP-positive solid tumors, including metastatic HR+, HER2−, HER2+, and TNBCs.

### 4.4 Gastrin-releasing peptide receptor (GRPR)

In normal physiology, the gastrin-releasing peptide receptor (GRPR) is activated by gastrin-releasing peptide and plays a role in the central nervous and gastrointestinal systems [94]. GRPR is frequently overexpressed in many solid tumors, including in more than 80% of primary and metastatic BCs [76], [95]. Binding of gastrin-releasing peptide to GRPR promotes BC-cell proliferation [96]. Preclinical data suggest that GRPR antagonists are preferable over agonists as radiopharmaceutical ligands, as they have higher selectivity for GRPR, higher tumor-to-kidney ratios, and are less prone to degradation compared with agonists. GRPR antagonists have greater plasma stability and are associated with fewer off-target effects that can lead to gastrointestinal toxicity due to GRPR activation in normal tissue [97].

A first-in-human phase 1 trial is investigating the safety and efficacy of the α-particle-generating, GRPR-targeting peptide ^212^Pb-DOTAM-GRPR1 in adults with HR+, HER2– MBC (NCT05283330). OMNIRAY (NCT07114601) is a phase 1 trial investigating the safety, tolerability, and efficacy of the radiopharmaceutical LY4257496, a novel GRPR antagonist radiolabeled with ^177^Lu, with or without standard-of-care therapy, in GRPR-positive (GRPR+) patients with advanced solid tumors, including HR+ MBC. Three studies are investigating the β^−^-emitting, GRPR-targeted peptide ^177^Lu-NeoBOMB1 (^177^Lu-NeoB) (NCT03872778), in combination with a cyclin-dependent kinase type 4 or 6 inhibitor (NCT05870579) or with chemotherapy (NCT06247995), in GRPR+, HR+, HER2– MBC. These three studies use a theranostic approach where GRPR expression is evaluated with ^68^Ga-NeoB imaging. Finally, recruitment for a phase 1 trial (NCT07661641) that will investigate the safety, efficacy, and dosimetry of the GRPR-targeted peptides A9-0631 and A9-0642 conjugated to the β-emitting ^177^Lu and α-emitting actinium-255 (^255^Ac), respectively, in patients with GRPR+, locally advanced, unresectable, or metastatic HR+, HER2– BC has started.

### 4.5 Bone metastasis (bone-targeting agents)

Bone metastases occur in approximately 70% of patients with MBC, causing considerable morbidity. Calcimimetic radiopharmaceutical therapies mimic the action of calcium and are incorporated into the bone matrix. Early studies demonstrated that radiopharmaceuticals were effective in alleviating pain from bone metastases (Table 2) [98], [99], [100]. Ortho-[^32^P]-phosphate was initially utilized in this context, but clinical utility was limited due to severe bone marrow suppression [101], [102], [103]. The next generation of therapeutic radiopharmaceuticals included the β-emitters strontium-89 chloride (^89^SrCl) and samarium-153 lexidronam (^153^Sm-EDTMP; NCT00365105), which were associated with pain relief in up to 80% of patients with mild-to-moderate bone marrow suppression [104], [105], [106], [107]. However, antecedent myelosuppressive chemotherapy that results in limited bone marrow reserve prohibits the use of these agents in clinical practice. The focus of ongoing BC trials with bone metastases treatment has shifted to the α-emitting radium-223 dichloride (^223^RaCl_2_; NCT04090398; alone or in combination with ET or chemotherapy) [108], [109], [110], [111], [112]. ^223^Ra improved symptomatic skeletal event-free survival (hazard ratio of 0.809 for ^233^Ra plus ET vs placebo plus ET) in patients with MBC; however, bone marrow suppression led to early termination of some studies [110]. ^223^Ra is not recommended for the treatment of MBC bone metastases because preserved bone marrow function is needed in these patients, who generally require cytotoxic chemotherapy [113].

### 4.6 Prostate-specific membrane antigen (PSMA)

PSMA, a type II membrane glycoprotein, is expressed in the endothelium of neovasculature in primary and MBCs, and is being investigated as a potential target in MBC trials [114]. The small-molecule PSMA-targeting ligand vipivotide tetraxetan conjugated to ^177^Lu (^177^Lu-PSMA-617) reduced tumor viability and angiogenesis in TNBC preclinical models [115], [116]. The potential of PSMA as a treatment target for MBC has recently been demonstrated by the prospective phase 2 PRISMA trial (NCT06059469) that identified PSMA expression with ^68^Ga-PSMA-11 in the tumor-associated vasculature of 93.8% of evaluated patients with metastatic TNBC. Analysis of 106 FDG-avid tumor lesions showed that 71 tumor lesions (67%) had PSMA uptake above that of liver background levels; however, individual patient results showed heterogeneity, as most patients (65%) had at least one lesion with PSMA uptake below liver background levels. Notably, PSMA immunohistochemistry showed limited discriminatory value [117]. Results from a phase 1 trial investigating the uptake and efficacy of the PSMA-targeting monoclonal antibody, ^177^Lu-J591 (NCT00967577) in various cancers, including MBC, are awaited. An ongoing phase 2 trial is investigating the safety and efficacy of the small-molecule PSMA-targeting ligand PSMA zadavotide guraxetan conjugated to the β^−^-emitting ^177^Lu (also known as ^177^Lu-PSMA Imaging & Therapy or ^177^Lu-PSMA I&T) (NCT05867615) in PSMA+ metastatic solid tumors.

### 4.7 Integrins

Integrins are heterodimeric transmembrane glycoproteins comprising α and β subunits that are involved in cell adhesion [118], [119]. These proteins play central roles in cancer angiogenesis, migration, cell proliferation, gene expression, survival, and motility, and enhanced expression correlates with metastatic potential and poor prognosis [118], [120]. Upregulation of integrins, including ανβ3, ανβ5, and ανβ6, in some cancers and in most tumor vasculature, along with low levels of expression in normal endothelial cells, has led to interest in the integrins as therapeutic targets [121], [122]. Integrin αvβ3 has been identified as a central modulator of treatment resistance to tyrosine kinase inhibitors, including in HER2+ MBC [123], [124]. An ongoing trial is assessing the safety and efficacy of ^177^Lu-DOTA-glu-integrin antagonist carbamate (^177^Lu-DOTAGO-IAC) (NCT04469127) in patients with angiogenic MBC (angiogenic protein expression pattern confirmed by immunohistochemistry). Another early-phase study is investigating the efficacy of [^177^Lu]Lu-albumin binder-pegylated cyclic region-glycine-di-aspartic acid dimer (^177^Lu-AB-3PRGD2) in any αvβ3-positive metastatic cancer (NCT05013086).

### 4.8 Other emerging targets

Overexpression of the transmembrane protein, carbonic anhydrase IX (CAIX), has emerged as a marker for hypoxia and poor prognosis in MBC [125], [126]. CAIX is currently under intense study for diagnostic imaging and therapy in clear-cell renal cell carcinomas, where it is often overexpressed [127]. CAIX facilitates maintenance of intracellular pH, while dysregulation increases intratumoral acidosis, and inhibiting CAIX decreases invasion, migration, and BC-cell survival [128], [129], [130]. An ongoing trial in patients with metastatic solid tumors, including TNBC, is evaluating the efficacy and safety of the small cyclic peptide CAIX-targeting radiopharmaceutical ^177^Lu-DPI-4452 after confirming CAIX expression with PET imaging using [^68^Ga]Ga-DPI-4452 (NCT05706129).

The cell adhesion molecule nectin-4 has been implicated in BC migration and metastasis, and it modulates antitumor immunity and drug resistance [131]. Nectin-4 expression further contributes to the pathogenesis of TNBC by increasing cancer stemness and decreasing OS [131], [132]. A first-in-human study is currently recruiting patients to determine the safety and efficacy of the nectin-4-targeted ligand α-emitting ^225^Ac-AKY-1189 in patients with solid tumors, including HR+ BC and TNBC (NCT07020117). Another early phase 1 trial (NCT07439120) is recruiting patients with advanced MBC (preferably following urothelial cancer) to investigate the safety, pharmacokinetics, and dosimetry of the first-in-class, β-emitting, Nectin-4-targeted peptide drug conjugate [^177^Lu]^177^Lu-BRP-020063 (^177^Lu-BRP-020063).

B7-H3 is also emerging as a promising target, with low expression in healthy cells, but high expression in the stromal cells of the tumor microenvironment. Under normal physiologic conditions, B7-H3 mainly has a role in the immune system, downregulating adaptive immunity by inhibiting T-cell activation and proliferation [133]. In cancer cells, B7-H3 is associated with protumorigenic effects on tumor cell metabolism, including increasing migration, invasion, angiogenesis, and chemoresistance [133]. A phase 1/2a study is investigating the β^−^-emitting ^177^Lu-anti-B7-H3 monoclonal antibody (^177^Lu-BetaBart) in patients with relapsed/refractory, locally advanced inoperable, or metastatic solid tumors, including patients with TNBC (NCT07189871).

Overexpression of programmed death ligand 1 (PD-L1) allows TNBC to escape immune cell destruction, and is linked to poor prognosis [134], [135]. Anti-PD-L1 and anti-PD1 antibodies, combined with chemotherapy, improve the survival of patients with early-stage and PD-L1-positive metastatic TNBC [36]. An ongoing study is investigating the safety and antitumor activity of ^177^Lu-RAD204, a single-domain anti-PD-L1 antibody conjugated to the β^−^-emitting radionuclide ^177^Lu, in patients with metastatic solid tumors, including TNBC (NCT06305962).

Cholesterol accumulation as lipid rafts within cancer and immune cell membranes facilitates cell adhesion, migration, survival, immune responses, and the development of drug resistance [136], [137], [138]. The radiolabeled phospholipid ether analog, iodine-131-iopofosine (^131^I-CLR1404), which is selectively absorbed and retained within lipid rafts, has been evaluated in two phase 1 clinical trials (NCT01495663 and NCT00925275) in which one patient with metastatic TNBC showed prolonged stable disease [139], [140]. Another phase 1 trial is recruiting patients with TNBC to determine the efficacy and safety of the auger-emitting phospholipid ether analog iodine-125-CLR125 (^125^I-CLR125) (NCT07311993).

Tumor-associated breast epithelial mucin is overexpressed in a subset of BCs and is associated with tumor aggressiveness and poor prognosis [141]. The radiolabeled murine immunoglobulin G, ^90^Y-BrE-3 (NCT00007891), which binds to epithelial glycoprotein mucin antigens on tumor cells, showed successful tumor localization in a phase 1 trial [142], [143]. Early efficacy results in six patients with MBC showed that half of the patients had an objective response after a single dose, with favorable safety [144].

## 5 Clinical management and administration of radiopharmaceutical therapy

Radiopharmaceutical therapies are generally delivered as repeated administrations (cycles) of a defined administered activity to optimize the radiation dose delivered to tumor cells while providing normal tissues with time to repair sublethal damage [4], [5], [145]. Despite this, significant myelosuppression limited the utilization of historical radiopharmaceutical calcimimetic therapies, which mimic the action of calcium. Early trials of these agents included heavily pretreated metastatic patients with compromised bone marrow reserve, who received radionuclides with higher LET and deeper tissue penetration [146]. Additionally, patients treated with ^89^SrCl or ^153^Sm-EDTMP, which have high bone uptake, commonly developed transient thrombocytopenia and neutropenia, which were manageable with dose reduction [146], [147], [148]. These results demonstrated that the myelosuppressive toxicity associated with some radionuclide therapies is dose-dependent [146].

Administration of radiopharmaceutical therapy in clinical practice requires careful monitoring of hematologic, renal, and hepatotoxicities [149]. Current recommendations by the International Commission on Radiological Protection (ICRP) prohibit radiopharmaceutical therapy during pregnancy and breastfeeding [150]. Women of child-bearing potential who may seek to become pregnant are advised to prevent conception during treatment and delay pregnancy for several months after treatment completion [150]. The majority of therapeutic agents have renal excretion, and recommendations regarding management of urine waste, hygiene, and a short period of isolation in the initial few days after administration are important, with increasing restrictions with higher administered doses or treatments with a longer half-life [150]. It is also recommended that patients limit contact with young children for a few days post-administration of radiopharmaceuticals [150].

Healthcare providers working with radiopharmaceuticals need to take precautions to limit radiation exposure, and most facilities follow the three core ALARA (**a**s **l**ow **a**s **r**easonably **a**chievable) principles, i.e. time, distance, and shielding. These principles suggest reducing the time spent near the radiation source, increasing the space between individuals and the radiation source, and placing physical barriers between individuals and the radiation source [151].

Radionuclide half-life influences radiation safety and distribution logistics during the production and delivery of radioligands, as well as radioactive waste management [152]. For instance, the short half-lives of some radionuclides obviate the need for long-term storage, but synchronized scheduling and coordination between production and delivery of these products are essential for the success of radiopharmaceutical imaging and treatment facilities [153], [154].

Radiopharmaceutical therapy delivery requires collaboration and training across multidisciplinary teams to ensure optimal patient care and access to service referral networks, with particular attention to the roles of the nuclear medicine physician or radiation oncologist, medical oncologist, medical physicist, and radiation safety officer [155], [156], [157], [158], [159]. Considerable infrastructure investment is required in generating and delivering molecular imaging and treatment agents, and in subsequent management of radioactive waste, although many large medical centers and increasing numbers of community cancer centers already bear this infrastructure [154], [160], [161], [162], [163]. Regulatory and reimbursement frameworks, including standardized patient care pathways, will further increase access to radiopharmaceuticals, and key regulatory agencies are in the process of providing guidance on the clinical evaluation of radiopharmaceutical therapies [155], [164], [165]. Furthermore, best practices that have been developed for radiopharmaceutical therapy administration in other solid tumors can be adopted for BC [153], [154], [164].

## 6 Future perspectives

Radiopharmaceutical therapy generally relies on imaging-informed confirmation of target expression prior to therapy administration. In the future, imaging may be complemented or replaced by tissue immunohistochemistry to assess target expression as each method has distinct strengths. While immunohistochemistry can capture cellular histopathology, radiopharmaceutical imaging informs whole-body disease expression and lesion heterogeneity. Radiopharmaceutical imaging will retain value in non-invasively assessing disease heterogeneity and when biopsy for immunohistochemistry is difficult or inconclusive [166], [167]. Furthermore, since radiopharmaceutical imaging can inform spatial and temporal changes, serial images can potentially be used to monitor disease progression and treatment response [168]. Routine adoption of radiopharmaceutical imaging is dependent on access to facilities capable of performing nuclear imaging and standardizing imaging methodology.

As the clinical utility of radiopharmaceutical therapy in BC expands, combination therapies will be pursued to enhance clinical efficacy [169], [170], [171], [172]. Examples include agents that increase cancer cell binding of radiopharmaceuticals, improve radiosensitization by inhibiting DNA damage repair mechanisms, or potentiate radiation-induced antitumor immunity [169], [173], [174]. Also, how to sequence radiopharmaceutical therapy within the increasingly complex treatment landscape of MBC will be an important focus of future trials [172]. Since the main mechanism of action for radiopharmaceutical therapies is DNA damage, combinations with other approved systemic BC treatments can potentially increase its efficacy [169]. The addition of chemotherapy may increase DNA damage while the addition of poly (ADP-ribose) polymerase or mechanistic target of rapamycin inhibitors could potentially increase radiosensitization by inhibiting cellular repair pathways [169]. Preclinical studies have shown that immune checkpoint inhibitors in combination with radiopharmaceutical therapy upregulate tumor-associated antigens, including PD-L1 [175], [176]. Adoption of radiopharmaceutical combinations is dependent on an increased understanding of radiobiology and mitigation of potential cumulative toxicities. While safety remains a concern, radiopharmaceutical therapies could also be considered earlier in the disease course when patients are naïve to other systemic treatments (i.e. before bone marrow depletion or other treatment-related toxicities have occurred). Furthermore, combination strategies that increase the efficacy of radiopharmaceuticals may potentially decrease the dosage and cycles of treatment needed, potentially reducing short- and long-term toxicities.

## CRediT authorship contribution statement

**Joyce O'Shaughnessy:** Writing – review & editing, Writing – original draft, Project administration, Conceptualization. **Neil K. Taunk:** Writing – review & editing, Writing – original draft, Project administration, Conceptualization. **Gary A. Ulaner:** Writing – review & editing, Writing – original draft, Project administration, Conceptualization.

## Declaration of competing interests

The authors declare the following financial interests/personal relationships which may be considered as potential competing interests: Joyce O'Shaughnessy reports article publishing charges and writing assistance were provided by Novartis Pharmaceuticals Corporation. Joyce O'Shaughnessy reports a relationship with Novartis that includes: consulting or advisory and travel reimbursement. Joyce O'Shaughnessy reports a relationship with Pfizer that includes: consulting or advisory. Joyce O'Shaughnessy reports a relationship with Eli Lilly that includes: consulting or advisory and travel reimbursement. Joyce O'Shaughnessy reports a relationship with AstraZeneca that includes: consulting or advisory. Joyce O'Shaughnessy reports a relationship with Eisai that includes: consulting or advisory. Joyce O'Shaughnessy reports a relationship with Agendia that includes: consulting or advisory. Joyce O'Shaughnessy reports a relationship with Amgen that includes: consulting or advisory. Joyce O'Shaughnessy reports a relationship with Genentech that includes: consulting or advisory. Joyce O'Shaughnessy reports a relationship with Merck that includes: consulting or advisory and travel reimbursement. Joyce O'Shaughnessy reports a relationship with Puma Biotechnology Inc that includes: consulting or advisory. Joyce O'Shaughnessy reports a relationship with Roche that includes: consulting or advisory. Joyce O'Shaughnessy reports a relationship with Sanofi that includes: consulting or advisory. Joyce O'Shaughnessy reports a relationship with Daiichi Sankyo that includes: consulting or advisory and travel reimbursement. Joyce O'Shaughnessy reports a relationship with Aptitude Health that includes: employment and travel reimbursement. Joyce O'Shaughnessy reports a relationship with G1 Therapeutics Inc that includes: consulting or advisory. Joyce O'Shaughnessy reports a relationship with Gilead Sciences that includes: consulting or advisory. Joyce O'Shaughnessy reports a relationship with Pierre Fabre that includes: consulting or advisory. Joyce O'Shaughnessy reports a relationship with Seagen that includes: consulting or advisory. Joyce O'Shaughnessy reports a relationship with BioNTech that includes: consulting or advisory. Joyce O'Shaughnessy reports a relationship with DAVA Oncology that includes: consulting or advisory and travel reimbursement. Joyce O'Shaughnessy reports a relationship with Stemline Therapeutics that includes: consulting or advisory. Joyce O'Shaughnessy reports a relationship with AADi that includes: consulting or advisory. Joyce O'Shaughnessy reports a relationship with Bristol-Myers Squibb that includes: consulting or advisory. Joyce O'Shaughnessy reports a relationship with Duality Biologics Ltd that includes: consulting or advisory. Joyce O'Shaughnessy reports a relationship with Ellipses Pharma Limited that includes: consulting or advisory. Joyce O'Shaughnessy reports a relationship with Exact Sciences that includes: consulting or advisory. Joyce O'Shaughnessy reports a relationship with Guardant Health that includes: consulting or advisory. Joyce O'Shaughnessy reports a relationship with Hiber Cell that includes: consulting or advisory. Joyce O'Shaughnessy reports a relationship with Jazz Pharmaceuticals that includes: consulting or advisory. Joyce O'Shaughnessy reports a relationship with J&J that includes: consulting or advisory. Joyce O'Shaughnessy reports a relationship with Menarini Stemline that includes: consulting or advisory. Joyce O'Shaughnessy reports a relationship with Mersana Therapeutics Inc that includes: consulting or advisory. Joyce O'Shaughnessy reports a relationship with Natera that includes: consulting or advisory. Joyce O'Shaughnessy reports a relationship with Summit Therapeutics that includes:. Joyce O'Shaughnessy reports a relationship with Tempus that includes: consulting or advisory. Joyce O'Shaughnessy reports a relationship with Tersera that includes: consulting or advisory. Neil Taunk reports article publishing charges and writing assistance were provided by Novartis Pharmaceuticals Corporation. Neil K. Taunk reports a relationship with Boston Scientific Corporation that includes: consulting or advisory. Neil K. Taunk reports a relationship with Novartis that includes: consulting or advisory and funding grants. Neil K. Taunk reports a relationship with Eli Lilly that includes: consulting or advisory. Neil K. Taunk reports a relationship with Varian Medical Systems that includes: consulting or advisory and funding grants. Neil K. Taunk reports a relationship with GE Healthcare that includes: consulting or advisory. Neil K. Taunk reports a relationship with TheraPanacea that includes: funding grants. Neil K. Taunk reports a relationship with RayzeBio, Inc. that includes: funding grants. Gary A. Ulaner reports article publishing charges and writing assistance were provided by Novartis Pharmaceuticals Corporation. Gary A. Ulaner reports a relationship with GE Healthcare that includes: consulting or advisory, funding grants, and speaking and lecture fees. Gary A. Ulaner reports a relationship with Lantheus that includes: consulting or advisory, funding grants, speaking and lecture fees, and travel reimbursement. Gary A. Ulaner reports a relationship with NUCLIDIUM that includes: consulting or advisory. Gary A. Ulaner reports a relationship with Curium US LLC that includes: consulting or advisory. Gary A. Ulaner reports a relationship with Novartis that includes: funding grants. Gary A. Ulaner reports a relationship with Eli Lilly that includes: funding grants. Gary A. Ulaner reports a relationship with Aktis Oncology, Inc. that includes: funding grants. Gary A. Ulaner reports a relationship with Abdera Therapeutics Inc. that includes: funding grants. Gary A. Ulaner reports a relationship with RayzeBio, Inc. that includes: funding grants. Gary A. Ulaner reports a relationship with AstraZeneca that includes: funding grants. If there are other authors, they declare that they have no known competing financial interests or personal relationships that could have appeared to influence the work reported in this paper.
